# Parental warmth interacts with several genes to affect executive function components: a genome-wide environment interaction study

**DOI:** 10.1186/s12863-020-0819-8

**Published:** 2020-02-04

**Authors:** Chunhui Chen, Chuansheng Chen, Gui Xue, Qi Dong, Libo Zhao, Shudong Zhang

**Affiliations:** 1 State Key Laboratory of Cognitive Neuroscience and Learning & IDG/McGovern Institute for Brain Research, Beijing Normal University, Beijing, China; 2 Beijing Key Laboratory of Brain Imaging and Connectomics, Beijing Normal University, Beijing, China; 3 Department of Psychological Science, University of California, Irvine, CA USA; 4 Department of Psychology, BeiHang University, Beijing, 100191 China; 5 Faculty of Education, Beijing Normal University, Beijing, China

**Keywords:** Executive function, Parental warmth, Gene set enrichment analysis, Gene-environment interaction

## Abstract

**Background:**

Executive function (EF) is vital to human beings. It has been linked to many genes and family environmental factors in separate studies, but few studies have examined the potential interactions between gene(s) and environmental factor(s). The current study explored the whole genome to identify SNPs, genes, and pathways that interacted with parental warmth (PW) on EF.

**Results:**

Nine EF tasks were used to measure its three components (common EF, updating, shifting) based on the model proposed by Miyake et al. (2000). We found that rs111605473, *LAMP5, SLC4A7,* and *LRRK1* interacted significantly with PW to affect the updating component of EF, and the GSE43955 pathway interacted significantly with PW to affect the common EF component.

**Conclusions:**

The current study is the first to identify genes that interacted with PW to affect EF. Further studies are needed to reveal the underlying mechanism.

## Background

Executive function (EF) is a set of general-purpose control processes that regulate thoughts and behaviors [1]. Its neural and genetic bases have been studied extensively. For example, a recent review concluded that the medial prefrontal and orbital frontal cortices are involved in different aspects of EF, and that genes related to neurotransmitters (i.e., dopamine, serotonin, norepinephrine, and acetylcholine) modulate functions of these brain regions and hence contribute to EF [2]. Although an earlier genome-wide association study (GWAS) failed to find genome-wide significant results for EF [3], later GWASs have identified many genes that are associated with EF measured by various tasks, including *CADM2* with the letter-digit or digit-symbol substitution task [4], *DSG1* with the trail making task [5], *WDR72* with the D-KEFS inhibition test [6], *RNASE13* with the ADNI-1 neuropsychological battery EF test [7], and *B3GNT7* and *NCL* with the antisaccade task [8].

Many social/family environmental factors have also been correlated with EF, such as stressful life events, maternal substance abuse during pregnancy, family socio-economic status, parental mental health, parenting practices, and parental warmth (PW) [9–13]. Harsh parenting was found to be associated with poor EF in children [10, 13, 14], perhaps because environmental stress acts through the HPA axis’s activity to influence behavior [9]. Indeed, harsh parenting or low PW has been linked to higher cortisol response [15] and poorer EF. However, how parenting or PW specifically interacts with genes to affect EF is not well understood. Thus far, only two candidate gene studies have found that *ANKK1* [16] and *COMT* [17] interact with parenting to affect children’s EF. No GWAS, or rather Genome-Wide Environment Interaction studies (GWEIS), has been conducted on EF. Following the procedures used in other GWEIS [18–20], the current study examined how genes (gene sets or genetic pathways) interacted with PW to affect EF. We adopted the widely used Miyake model of EF with three components: updating (constant monitoring and rapid addition / deletion of working memory contents), shifting (switching flexibly between tasks or mental sets), and common EF [1, 21]. We also used a parental warmth questionnaire that has been found to interact with genes to affect decision making [22].

## Results

### Behavioral performance

**Fig. 1:**
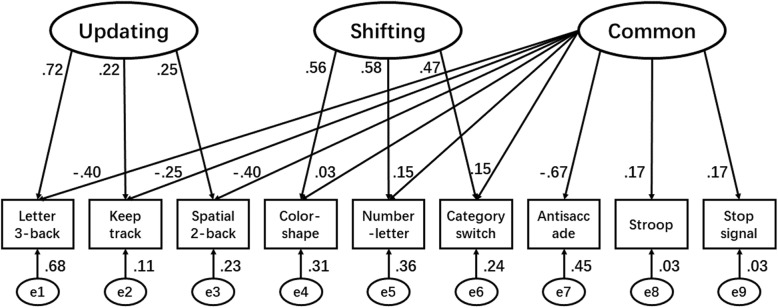
Confirmatory factor analysis of the componential executive function model

Table 1 shows the means and standard deviations (SD) of all nine EF tasks as well as their bivariate correlations. As expected, each set of 3 tasks measuring the same component were highly inter-correlated. In addition, the antisaccade task was correlated with the 3 updating tasks and the Stroop task was correlated with the 3 shifting tasks. These data fit Miyake’s EF model well (Fig. 1), χ^2^(21) = 52.738, *p* < 0.001, RMSEA = 0.033, NFI = 0.935, IFI = 0.960, TLI = 0.911, CFI = 0.958, all suggesting good model fit. We thus calculated the component scores using AMOS for the following gene-environment interaction analysis.

**Table 1:** Descriptive statistics and bivariate correlations of the main study variables

	Letter 3-back	Keep track	Spatial 2-back	Color shape	Number letter	Category switch	Anti saccade	Stroop	Stop signal	Updating	Shifting	Common
Mean	0.80	30.79	0.90	0.25	0.24	0.26	0.74	0.13	195.39	0.42	0.21	0.58
SD	0.11	3.13	0.09	0.17	0.15	0.12	0.14	0.08	55.91	0.06	0.07	0.07
Correlation with Age (r)	−0.05	-4E-3	1E-3	− 0.07^*^	− 0.05	− 0.04	− 0.07^*^	0.02	0.08^*^	− 0.03	− 0.08^**^	− 0.06
Gender difference (T)	2.43^*^	0.52	3.34^***^	− 0.96	−2.88^**^	− 0.60	6.54^***^	0.18	0.41	0.79	−1.57	6.52^***^
Bivariate correlations												
Letter 3-back										0.96^**^	−0.03	0.51^**^
Keep track	.25^***^									0.27^**^	−0.06	0.34^**^
Spatial 2-back	.34^***^	.15^***^								0.33^**^	−0.04	0.56^**^
Color-shape	−.03	−.01	−.07^*^							−0.04	0.74^**^	−0.05
Number-letter	−.06^*^	−.08^**^	−.06^*^	.33^***^						2E-3	0.79^**^	−0.22^**^
Category switch	−.09^**^	−.05	−.08^**^	.26^***^	.29^***^					−0.04	0.63^**^	−0.19^**^
Antisaccade	.27^***^	.16^***^	.27^***^	.03	−.09^***^	−.08^**^				−0.04	0.03	0.90^**^
Stroop	−.02	.02	−.08^**^	.11^***^	.11^***^	.08^**^	−.11^***^			0.05	0.11^**^	−0.23^**^
Stop signal	−.05	−.07^*^	−.06^*^	.06	−.01	−.01	−.11^***^	.06^*^		−0.06	−0.03	− 0.15^**^

Note: * *p* < 0.05, ** *p* < 0.01, *** *p* < 0.001

Table 1 also shows age and gender differences in task performance. There were age effects on the color-shape and antisaccade tasks, and gender effects on the letter 3-back, spatial 2-back, number-letter, and antisaccade tasks. The shifting component of EF was negatively correlated with age, and the common EF component showed a gender difference, with males scoring higher than females. Age and gender were included as covariates in the following analyses.

### GWAS and gene/Geneset enrichment analysis

**Fig. 2:**
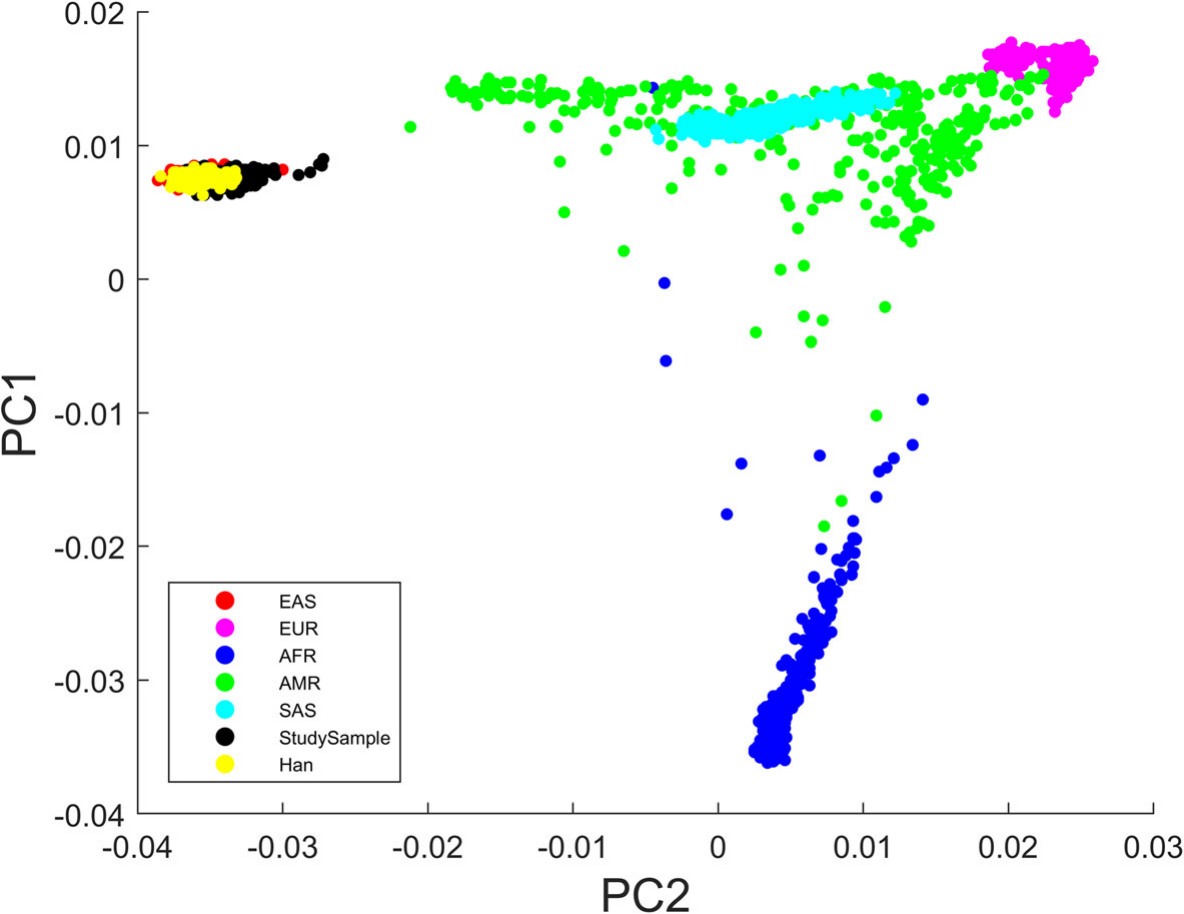
Projection of the current study samples onto the first two principal components inferred from the 1000 Genomes Project’s phase3 populations. The current study sample overlapped with East Asian (EAS) and Han Chinese populations

A principal component analysis (PCA) showed that the current sample overlapped with East Asian (EAS) and Han Chinese from the 1000 Genomes data, and clearly separated this sample from other populations (Fig. 2). This result suggests that this sample did not have a population stratification problem.

**Fig. 3:**
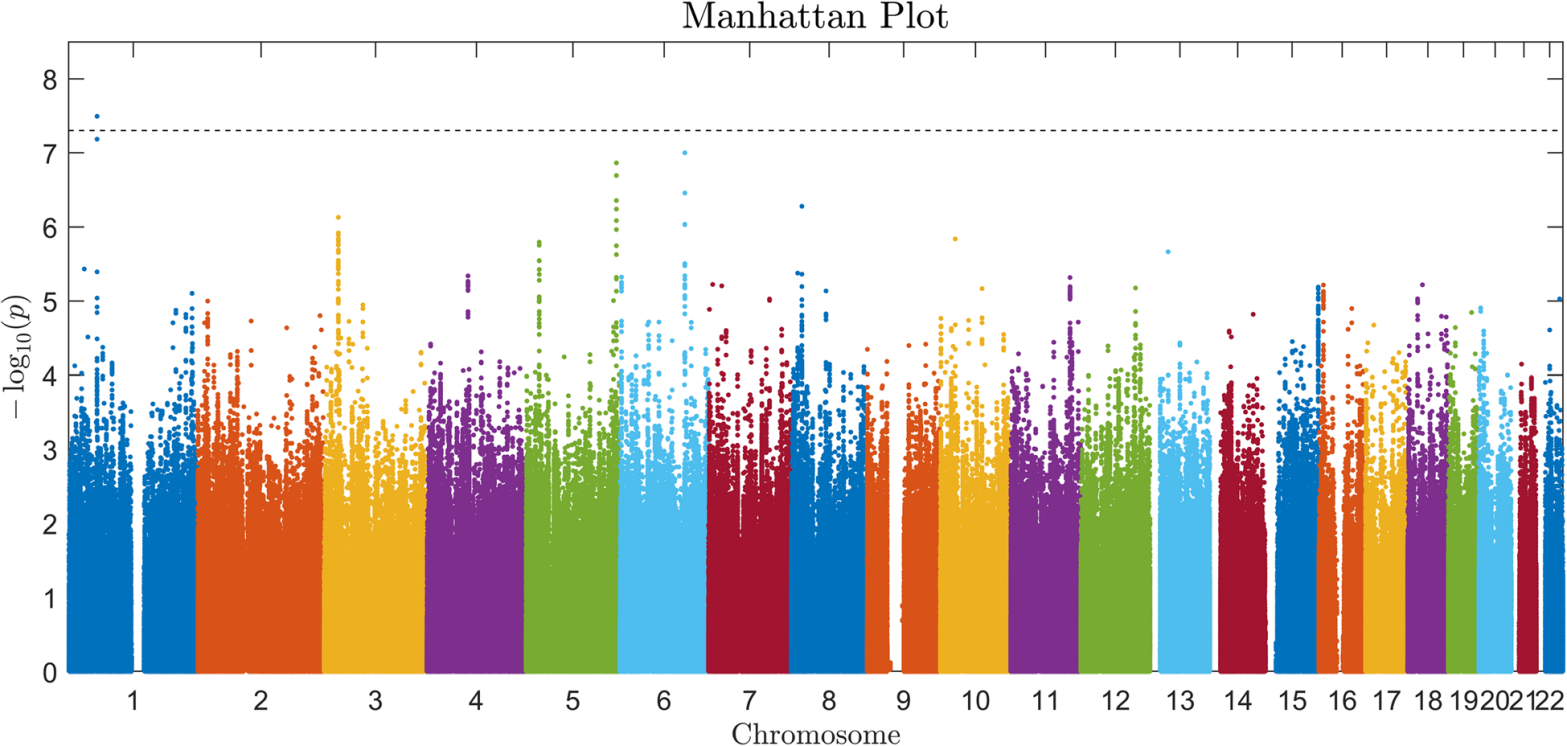
Manhattan plot of genome-wide interaction with PW for updating. Each dot represents the *p* value of a SNP-by-PW interaction. X axis shows the chromosomal positions of the SNPs, and Y axis shows –log10 transformed interaction *p* values. The dashed line represents *p* = 5E-8. Only one SNP at chromosome 1 survived this genome-wide significance threshold

**Fig. 4:**
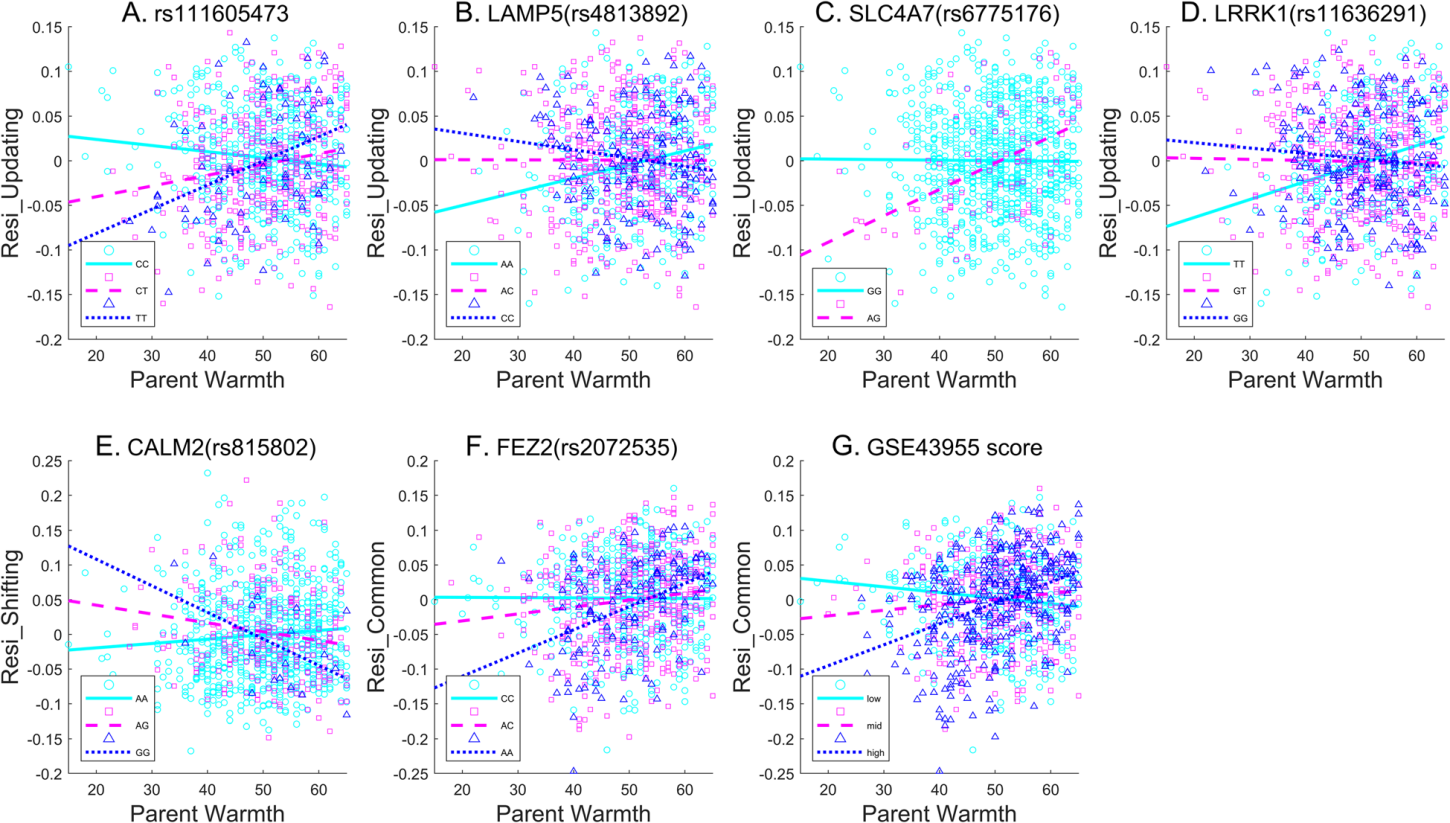
Interaction effects of representative SNPs listed in Table 2. The X axis represents PW and the Y axis represents EF components with age, gender, and first two principal components of genome regressed out, to keep consistent with GWEIS analysis. Each subplot A-G showed one interaction effect presented in Table 2, correlations between EF and PW are shown for different genotype groups

A whole-genome-by-PW interaction analysis was run for each of the three EF components. Using the genome-wide significance threshold (*p* < 5E-8), only updating had a significant interaction effect at rs111605473 (*p* = 3.208e-08) (Fig. 3). CC homozygotes showed a negative correlation between PW and updating, while T allele carriers (TT homozygotes and the heterozygotes) showed positive correlations (Fig. 4a and Table 2). This SNP is located on an inter-gene region of chromosome 1, about 50 kb downstream of *SSBP3* and 50 kb upstream of *LOC105378735*. The other two EF components did not have genome-wide significant interaction effects.

**Table 2:** Correlations between EF and PW for each genotype group based on the representative SNPs

SNPs	EF	genotype	r	p	n	genotype	r	p	n	genotype	r	p	n
rs111605473	Updating	CC	−0.11	0.015	522	CT	0.18	2.8E-04	405	TT	0.40	4.0E-04	75
LAMP5(rs4813892)	Updating	AA	0.22	3.0E-05	353	AC	3.0E-03	0.956	484	CC	−0.15	0.056	165
SLC4A7(rs6775176)	Updating	GG	−0.01	0.796	904	AG	0.50	1.8E-07	96	AA	-^a^	-^a^	2
LRRK1(rs11636291)	Updating	TT	0.29	6.7E-07	282	GT	−0.02	0.674	502	GG	−0.09	0.18	218
CALM2(rs815802)	Shifting	AA	0.09	0.020	727	AG	−0.17	0.005	257	GG	−0.52	0.028	18
FEZ2(rs2072535)	Common	CC	−0.01	0.909	417	AC	0.13	0.004	453	AA	0.42	5.6E-07	132
GSE43955 score	Common	low	−0.13	0.019	335	mid	0.11	0.054	333	high	0.38	5.6E-13	334

Note: Each row shows the details of a significant interaction (for a specific SNP and an EF component). A correlation (and associated p value and sample size) is shown for each genotype (in the order of major allele homozygotes, heterozygotes, and minor allele homozygotes, see each row for specific alleles)

^a^ not calculated because of only 2 participants

Gene level analysis revealed that the *LAMP5, SLC4A7,* and *LRRK1* genes significantly interacted with PW to affect updating after FDR correction (Table 3). Although the interaction effects for the shifting and common EF components did not survive FDR correction, the genes with the most significant results are also included in Table 3. These interaction effects are shown in Fig. 4b-f and Table 2 using the most significant SNP within each gene. AA homozygotes of rs4813892 within *LAMP5*, AG heterozygotes of rs6775176 within *SLC4A7*, and TT homozygotes of rs11636291 within *LRRK1* showed significant positive correlations between updating and PW, but the other genotype groups showed no significant correlations. AA homozygotes of rs815802 within *CALM2* showed a positive correlation between PW and shifting, while AG heterozygotes and GG homozygotes showed a negative correlation. AA and AC groups of rs2072535 within FEZ2 showed a positive correlation between PW and the common EF component, but CC homozygotes did not show a significant correlation.

**Table 3:** Genes that showed most significant interactions with PW to affect EF components

EF components	GENE	CHR	START	STOP	ZSTAT	Gene P	Top SNP
Updating	**LAMP5**	**20**	**9,495,005**	**9,511,171**	**4.3928**	**5.6E-06**	**rs4813892**
	**SLC4A7**	**3**	**27,414,212**	**27,525,911**	**4.2862**	**9.09E-06**	**rs6775176**
	**LRRK1**	**15**	**101,459,460**	**101,610,317**	**4.258**	**1.03E-05**	**rs11636291**
Shifting	CALM2	2	47,387,221	47,404,229	3.8548	5.79E-05	rs815802
Common	FEZ2	2	36,779,397	36,825,333	4.4018	5.37E-06	rs2072535

Note: Genes for updating survived FDR correction and are shown in bold

All these top SNPs are intronic. A search in the BrainSeq database (http://eqtl.brainseq.org/phase1/eqtl/) showed that rs6775176 is strongly associated with transcripts of NEK10 (minimum *p* = 7.5E-5, just 3 kb away from SLC4A7), with GG homozygotes showing the highest level of expression. SNP rs815802 is strongly associated with transcripts of CALM2 (minimum *p* = 3.1E-9), with AA homozygotes showing the highest level of expression. SNP rs2072535 is strongly associated with transcripts of FEZ2 (minimum *p* = 4.0E-27), with AA homozygotes showing the highest level of expression.

Pathway level analysis showed that one pathway had a significant interaction with PW to affect the common EF component after FDR correction (*p* = 2.08E-06). This pathway is involved in the immunologic system, consisted of 186 genes (http://www.broadinstitute.org/gsea/msigdb/cards/GSE43955_TH0_VS_TGFB_IL6_TH17_ACT_CD4_TCELL_60H_DN). We refer to this pathway as GSE43955 hereafter. To demonstrate this interaction effect, a gene score of this pathway was calculated. Genes within this pathway with nominal interaction effects (*p* < 0.05) were identified, and the most significant SNP within each gene was selected. The gene score was calculated by multiplying genotype (coded as 0/1/2 in terms of the number of copies of the minor allele) of these significant SNPs with their corresponding effect sizes (beta of the interaction term in PLINK results) and then summing them up. Participants were then equally divided into low, middle, and high gene score groups to illustrate the interaction effect. The low gene score group showed a significant negative correlation between PW and EF, the middle gene score group showed a marginally positive correlation, while the high gene score group showed a very significant positive correlation (Fig. 4g and Table 2).

## Discussion

This study confirmed the EF model proposed by Miyake and Friedman [1, 21, 23] with the extraction of three EF components (i.e., the common, updating, and shifting components) and identified SNPs, genes, and a pathway that interacted with PW to affect these components.

Parenting behaviors have been found to interact with genes to affect human traits like creativity [24], depression [25], aggression [26], externalizing behavior [27], self-control [28], as well as EF [16, 17]. Researchers have proposed that parenting behaviors likely influence the way children cope with stress, resulting in epigenetic changes in the HPA axis such as glucocorticoid receptor expression [29] or in the neurotransmitter systems such as dopamine, oxytocin, and serotonin [30]. All these studies, however, used only candidate genes. To the best of our knowledge, the current study is the first to search for interaction effects between PW using the whole genome. We found genes related to neural growth and function and the immunologic system that interacted with PW to affect EF.

Specifically, we found that rs111605473 significantly interacted with PW to affect updating. This SNP is located downstream of *SSBP3*, and hence possibly modulates the function of this gene. SSBP3 can bind to DNA and regulate transcription, and has been found to regulate mouse embryonic stem cells differentiating into trophoblast-like cells [31]. It is possible that this gene interacts with parenting behavior during early development and hence influences later EF. It is also possible that rs111605473 simply tags some other causal SNPs/genes in this region.

We found that *LAMP5, SLC4A7,* and *LRRK1* interacted with PW to affect updating. *LAMP5* is extensively expressed in mouse brain, can alter short term synaptic plasticity, and is involved in GABAergic transmission [32]. This is consistent with the facts that dynamic synaptic plasticity is vital for working memory (i.e., updating) [33] and that GABAergic interneurons in the prefrontal cortex are involved in EF [2]. *SLC4A7* has been associated with breast cancer and hypertension, but also has been proposed to influence neurotransmitter in the brain [34] and EF [2]. *LRRK1* is extensively expressed in human brain, especially in the hippocampus [35], and has been found to be a risk gene for Parkinson’s disease [36], suggesting the possibility that *LRRK1* acts on the function of the hippocampus to influence working memory and contributes to EF deficit associated with Parkinson’s disease.

The genes that showed the most significant interactions with PW to affect shifting and the common component of EF were *CALM2* and *FEZ2*, although they did not survive FDR correction. Both genes are expressed extensively in human brain. *CALM2* belongs to the calmodulin gene family. Calmodulin is a calcium binding protein found mainly in the central nervous system and is involved in signaling pathway and neuronal cell death [37]. *FEZ2* is important for axonal bundling and elongation.

In summary, all these genes have been linked to the nervous system although their function on cognition has seldom been tested. According to the BrainSeq database, the most significant SNPs within *SLC4A7, CALM2*, and *FEZ2* are shown to be relevant to their respective genes’ expression (in the case of the SNP for *SLC4A7*, it is relevant to the expression of the neighboring gene *NEK10*). The current study found that these genes interacted with PW to affect EF. It is likely that PW as an environmental factor can modulate these genes’ expression through some epigenetic processes. These interactions may also reflect genotypic differential susceptibility (i.e., individuals with certain genotypes are more susceptible to environmental influences [38, 39]). Further studies are needed to explicate the mechanisms involved.

Finally, we found that the GSE43955 pathway had a significant interaction with PW to affect the common EF component, although this pathway did not contain the significant SNPs/genes discussed above. This is perhaps due to the fact that many SNPs or genes have small effects that are not detectable at the SNP or gene level, but whose cumulative effect is robust and evident at the pathway level. This pathway contains the most often reported EF-related gene, the *APOE*. This gene had often been found to be associated with some aspects of cognition and brain disorders (i.e., Alzheimer’s disease). Our results suggest this gene and other immunologic genes may interact with PW together (i.e., gene score) to affect EF.

The sample size of the current study needs to be discussed. The sample size was not very large compared to that of recently published GWAS studies. Nevertheless, our study had enough power to detect moderate effects. Specifically, we did a power analysis using Gpower 3.1 [40]. We used F tests to test increased R^2^ by the interaction term in linear multiple regression, as modeled in PLINK. The effect size is defined as f^2^ = VS/VE, where VS is the proportion of variance explained by the interaction term, and VE is the error variance. The effect size of rs111605473-by-PW interaction was 0.03, so the power of finding this effect with a sample size of 1002 at α < 1E-7 (the minimum value Gpower can accept) was 0.59, and at the nominal α < 0.01 level the power was 0.99. Since MAGMA did not report effect sizes, we did a power analysis with the gene score of the significant gene set GSE43955. The effect size was 0.06, which was higher than those of single SNPs, as would be expected. The power at α < 1E-7 was 0.99, and that at α < 0.01 was 1. In conclusion, our sample size was sufficient to capture moderate gene-environment interactions, especially at the gene or pathway level.

Several limitations of the current study need to be mentioned. First, this study enrolled only healthy Chinese college students, whose results may not generalize to other populations or to samples with disorders. Second, our measure of environment (PW) was based on participants’ reports, which are subject to reporting biases. Future research should consider objective measures or at least an independent assessment by observers. Finally, the current study only revealed associations among genes, the environmental factor of PW, and EF. Further studies need to determine their causal relations and investigate the relevant biological mechanisms.

## Conclusions

In summary, the current study measured EF based on a reliable and widely accepted 3-component model, and found that rs111605473, *LAMP5, SLC4A7,* and *LRRK1* interacted significantly with PW to affect the updating component of EF, and that the GSE43955 pathway interacted significantly with PW to affect the common component of EF. In addition, *CALM2* and *FEZ2* interacted with PW to affect the shifting and common components, albeit at a lower level of significance. These results suggest that parenting’s effects on cognitive development may depend on the genetic makeup of the children. The specific mechanisms involved, however, need further investigation.

## Methods

### Participants

We enrolled healthy Chinese college students from Beijing Normal University in Beijing, China, and Southwest University in Chongqing, China. One thousand three hundred ninety-one participants (529 male and 862 female, age = 20.2+/− 1.8 years) completed the behavioral tests. One thousand two of them (401 male and 601 female, age = 20.4+/− 1.9 years) were genotyped. All participants were Han Chinese and reported no history of psychiatric diseases, head injuries, or stroke/seizure. Participants were paid for their participation.

### Behavioral measures

#### Parental warmth (PW)

Parental Warmth and Acceptance Scale [41] measures perceived parental warmth with 11 items, such as “My parents really understand me” and “My parents like me the way I am; they don’t try to ‘make me over’ into someone else”. Participants rated each item on a 6-point scale, 1 = “Disagree strongly” to 6 = “Agree strongly”. The total score of all items was used for analysis.

#### Executive function (EF)

Following the model of Miyake et al. [1, 21, 42], the current study used 9 EF tasks that were used in one of their original studies [21], with some modification in terms of materials or presentation parameters. These nine tasks measure the three latent components of EF: the keep track, letter 3-back, and spatial 2-back tasks were used to assess updating; the number-letter, color-shape, and category switch tasks were used to assess shifting, and all nine tasks (the above six plus the antisaccade, stop signal, and Stroop tasks) were used to assess the common EF component [1] (Fig. 1). We also used the same outcome index for each task as in Miyake’s model. A brief description of each task appears below.

##### Keep track

A list of 15 words were presented in the center of the screen one by one for 1.5 s each. The words belong to several categories and were presented in random order. Subjects had to memorize the last presented word of each category and write them down after the presentation. Six categories were used, including animals, colors, countries, distances, metals, and relatives. Twelve word lists were tested, with 4 lists containing words of 2 categories, 4 containing 3 categories, and 4 containing 4 categories. The total number of words correctly written down was used.

##### Letter 3-back

A sequence of 13 single letters were presented on the screen one by one, shown for 750 ms, followed by a blank screen of 2250 ms. Subjects had to memorize the latest 3 letters and judge if the current letter was the same as the one presented 3 items before. Subjects had to make response within 3 s as accurately and fast as possible. The whole task included 6 sequences. The overall accuracy of the 6 sequences was used. Before the formal test, subjects were given a practice session to familiarize themselves with the task. Practice ended if the subjects achieved an accuracy higher than 0.7 or practiced for 3 sequences, whichever came first, to avoid over-training.

##### Spatial 2-back

A sequence of 12 squares were presented one by one on the screen at random positions. Each square was presented for 0.5 s with an interval of 1.5 s between 2 squares. Subjects had to memorize the positions of the last two squares and judge if the current square was at the same position as the one 2 items before, and a response was required within 2 s. The whole task included 4 sequences. The overall accuracy was used. Before the formal test, subjects were given a practice session. Practice ended if the subjects achieved an accuracy higher than 0.7 or practiced for 3 sequences, whichever came first, to avoid over-training.

##### Number-letter

A number-letter pair (e.g. 7G) was shown in a rectangle on the screen. If the pair appeared on the upper side of the screen, participants judged if the number was odd or even by key pressing. If the pair appeared on the lower side, participants judged if the letter was a consonant or a vowel. Subjects had to respond as fast and accurately as possible. The stimuli disappeared immediately after the response. Stimuli were presented in pseudorandom order, resulting in equal numbers of trials with the same judgment task as the trial before it (the repeat condition) or with a different judgment task (the switch condition). Response time of the switch condition minus that of the repeat condition was used.

##### Color-shape

This task was similar to the number-letter task, but with different stimuli. A red or green circle or triangle was presented in the center of the screen, with a cue on top indicating whether participants should make judgment by color or by shape. Stimuli were also presented in pseudorandom order to ensure an equal number of repeat and switch trials. The response time difference between the two conditions was used.

##### Category switch

Similar to the number-letter task, a two-character Chinese word (i.e., 钥匙, key) was presented in the center of the screen, with a cue on top it. Participants had to judge according to the cue if the word describes a living or nonliving object, or if it is larger or smaller than a shoe case. Trials were presented in pseudorandom order to ensure an equal number of repeat and switch trials. The response time difference was used.

##### Antisaccade

A fixation “+” was presented for a duration randomly drawn from nine durations between 1.5 and 3.5 s in 0.25 s interval, followed by a 0.32 cm black square cue presented for 0.15 s on one side of the screen. Then a target of a 0.79 cm arrow within a 1.11 cm square was presented on the other side of the screen for 0.175 s and then masked by a grey square. Subjects had to control their attention not to the cue but to the target to identify the direction of the arrow by key pressing (left, up, right). Subjects practiced on 22 trials to learn the task, followed by 90 test trials. Accuracy of these 90 trials was used.

##### Stop signal

Participants were asked to press left or right button according to an arrow presented in the center of the screen for 1 s as accurately and quickly as possible (go trials). On 25% of trials, a red circle appeared around the arrow following the presentation of the arrow, participants had to withhold their response (nogo trials). This delay between the onset of the red circle and the arrow was adaptive based on task performance with an aim of 50% success at inhibiting responses during the nogo trials. The task consisted of 4 blocks, each with 64 trials. Stop-signal reaction time (SSRT) was calculated as the median response time of the go trials minus the mean delay of the nogo trials, using only trials with correct responses within the last two blocks.

##### Stroop

We adopted the classical Stroop task with 4 Chinese color words 红 (red), 绿 (green), 黄 (yellow), and 蓝 (blue). Each word was presented either in the color of the word’s meaning (i.e. the word “red” presented in red) under the congruent condition or in one of the other 3 colors (e.g., the word “red” presented in green) under the incongruent condition. Each condition had 12 trials, resulting in (12 congruent + 12 incongruent)*4 words = 96 trials, which were presented in random order. For each trial, a word was presented in the center of the screen, and participants had to respond to the printed color by pressing one of four keys as fast and accurately as possible. The response time difference between the two conditions was used as an index of inhibition. To make sure participants were familiar with the color-key association, they were first given a practice session, in which a color square was presented at the center of the screen and participants had to press a corresponding key quickly. The practice session ended when participants obtained an accuracy rate higher than 70%.

### Genotyping

1-2μg genome DNA (gDNA) was extracted from 250ul blood using Axypre Blood Genomic DNA Kit (Corning Life Sciences cat.no.11313KC3). The concentration of all gDNA was quantified with the Qubit2.0 Fluorometer (Life Technologies, cat. no. Q32866) and the Qubit dsDNA HS Assay Kit (Life Technologies, cat. no. Q32854). Six hundred twenty-nine samples were genotyped on the Infinium Human Omni-Zhonghua-8 chips, 239 samples were genotyped on the Infinium Human Omni2.5–8 exome chips, and 134 were genotyped on Infinium OminiExpress-12 chips (Illumina, San Diego, CA, USA), all according to the manufacturer’s specifications. Genotyping module of Genome studio v3.0 (Illumina, San Diego, CA, USA) was used to call the genotypes based on the fluorescent signal with standard cluster algorithm. Samples with call rate less than 98% (4 samples, with genotype call threshold of 0.15) were re-genotyped thus all passed this threshold in the final dataset. Further data cleaning was performed separately for each kind of chips, using PLINK2 (https://www.cog-genomics.org/plink/1.9/) [43, 44]: SNPs with missing data on more than 5% samples, or HWE *p* < 1E-6, or MAF < 0.01, were excluded, and subjects missing more than 5% SNPs were discarded too (no subjects were excluded by this threshold).

Autosome genotypes of 3 chips were then imputed separately using Michigan Imputation Server (https://imputationserer.sph.umich.edu/index.html) following their protocol: (1) HRC tools (www.well.ox.ac.uk/~wrayner/tools) were used to check strand and to flip to forward strand when necessary; (2) data were transformed to VCF files and sorted for each chromosome; (3) data were uploaded to the server, and imputed using 1000G Phase 3 EAS population as reference. Imputed data were cleaned using home-made codes, only SNPs with imputation quality r^2^ > 0.8 and MAF > 0.05 were retained. Then these datasets were merged and cleaned again (MAF > 0.05, HWE > 1E-6), retaining 4,856,474 SNPs. No duplicated or related subjects were identified (maximum PI_HAT = 0.0537, calculated with PLINK2).

To estimate the ancestry of our sample and determine whether we had a potential population stratification problem, we ran a principal component analysis on the 1000 Genomes Project’s data and projected our sample to the first two principal components using EIGENSTRAT software [45]. The 1000 Genomes Project’s phase3 data were downloaded from ftp://ftp.1000genomes.ebi.ac.uk/vol1/ftp/release/20130502/, converted into Plink format, combined with the current dataset. Overlapping SNPs were retained, cleaned with standard criteria (−-geno 0.05 --maf 0.02 --hwe 1e-6), pruned considering the EAS population (plink pruning: --indep-pairwise 200 5 0.25). Ambiguous strand SNPs were removed (AT or CG). A total of 105,831 SNPs remained for this analysis.

To further explore possible biological mechanism of the SNPs that showed the most significant results, we searched the BrainSeq Consortium database (http://eqtl.brainseq.org/phase1/eqtl/) for gene expression information. BrainSeq provides information about the associations between genotypes and RNA sequencing data collected from postmortem DLPFC tissues of 175 schizophrenia patients and 237 controls.

### Experimental procedure

The current study is part of a larger project that included extensive measures of executive function, decision making, memory, personality, and wellbeing. Tasks of different domains were interleaved and participants finished all tasks in the same order. It took about 4 h (2 h in the morning and 2 h in the afternoon) to complete all the tasks. To reduce the habituation and fatigue effects, each task was designed to be about 10 min long and if they would like to, participants were allowed to take a break after each task. A mandatory break was enforced after each hour. Blood sample were collected after the morning session and before lunch.

### Statistical analysis

Behavioral indices of the EF tasks were calculated. Values outside of 3 standard deviations for each index were treated as missing data. Latent EF components were modeled with IBM SPSS AMOS 22 using a nested factors model [21, 23]. That is, all nine tasks were used to define a common EF component; the keep tract, letter 3-back, and spatial 2-back tasks were used to extract the updating component; and the number-letter, color-shape, and category switch tasks were used to extract the shifting component.

Genome-wide environment interaction analysis was run using Plink2 linear regression, using each EF component as the dependent variable; PW, genotype and their interaction as independent variables; and age, gender, and first two principal components of the genome as co-variates. Interaction *p* values from Plink2 were inputted to MAGMA [46] for gene-set enrichment analysis. Gene definition was downloaded from the MAGMA website (https://ctg.cncr.nl/software/magma), using the NCBI37.3 version, resulting in 17,287 genes. The sum of –log(p) within a gene was calculated as the gene-level statistics (MAGMA default model). Seventeen thousand seven hundred seventy-nine gene sets from msigdb.v6.0 (http://software.broadinstitute.org/gsea/msigdb/) were used for pathway enrichment analysis. FDR correction was applied to the selection of significant genes and pathways.

## Data Availability

The datasets used and/or analyzed during the current study are available from the corresponding author upon reasonable request.

## Abbreviations

EF: Executive function
GWEIS: Genome-Wide Environment Interaction studies
PW: Parental warmth
